# Observed versus expected morbidity and mortality in patients undergoing mitral valve repair

**DOI:** 10.1093/icvts/ivac241

**Published:** 2022-10-07

**Authors:** Paige Newell, Richard Tartarini, Sameer Hirji, Morgan Harloff, Siobhan McGurk, Olena Cherkasky, Tsuyoshi Kaneko

**Affiliations:** Division of Cardiac Surgery, Brigham and Women’s Hospital, Harvard Medical School, Boston, MA, USA; Albany Medical College, Albany, NY, USA; Division of Cardiac Surgery, Brigham and Women’s Hospital, Harvard Medical School, Boston, MA, USA; Division of Cardiac Surgery, Brigham and Women’s Hospital, Harvard Medical School, Boston, MA, USA; Division of Cardiac Surgery, Brigham and Women’s Hospital, Harvard Medical School, Boston, MA, USA; Division of Cardiac Surgery, Brigham and Women’s Hospital, Harvard Medical School, Boston, MA, USA; Division of Cardiac Surgery, Brigham and Women’s Hospital, Harvard Medical School, Boston, MA, USA

**Keywords:** Mitral valve repair, Low risk, Morbidity and mortality

## Abstract

**OBJECTIVES:**

Mitral valve repair (MVP) is the gold standard treatment for degenerative mitral regurgitation. With the expansion of transcatheter technologies, this study compares the outcome of MVP in low-risk and non-low-risk patients to serve as a benchmark.

**METHODS:**

This retrospective, single-institution study examined all patients who underwent MVP for primary mitral regurgitation from 2005 to 2018. Patients were stratified into 2 risk categories: low-risk [Society of Thoracic Surgeons (STS) Predicted Risk of Mortality (STS-PROM) ≤2%] and non-low risk (STS-PROM > 2% or age > 75), with a subgroup of very low risk (STS-PROM ≤1%, age <75).

**RESULTS:**

A total of 1207 patients were included, and 1053 patients were classified as low risk and 154 as non-low risk. The non-low-risk group was significantly older, more likely to be female, and had a higher comorbidity burden than the low-risk group (all *P* < 0.01). For the low-risk group, the observed-to-expected (O:E) STS mortality ratio was 0.4 and the composite morbidity and mortality ratio was 0.6, whereas for the non-low risk, the O:E mortality was 1.5 and the composite morbidity and mortality was 0.9. When the subgroup of very low-risk group was assessed, the mortality O:E ratio was 0.

**CONCLUSIONS:**

The observed composite morbidity and mortality of patients undergoing MVP were persistently lower in low-risk patients, mainly driven by the very low-risk group. The excellent outcome of MVP in low-risk patients should be validated on a national level to determine how transcatheter technologies can be utilized in these patients.

**Figure:**
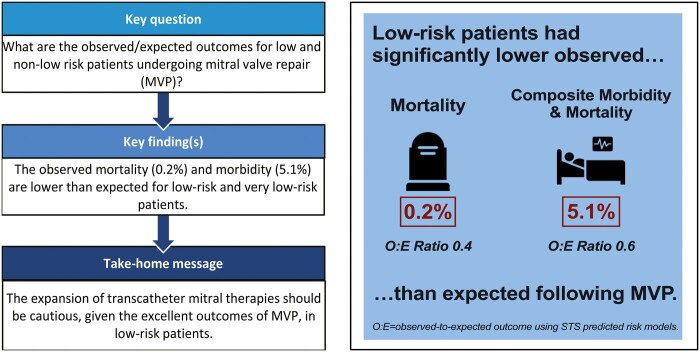

## INTRODUCTION

Mitral regurgitation (MR) is the most common valvular disease in the USA, with the burden expected to double by 2030 due to the ageing population [1]. For degenerative MR, surgical mitral valve repair (MVP) leads to increased quality of life and survival advantage [2–4]. Hence, the recently updated American College of Cardiology and American Heart Association 2020 valve guidelines recommend MVP over mitral valve replacement when the anatomic cause of MR is degenerative disease [5]. On the other hand, the landscape of cardiac surgery is continuously adapting to the advent of new technologies—particularly the less-invasive transcatheter devices. Although MVP remains the gold standard procedural option for patients with primary MR [6, 7], transcatheter edge-to-edge repair (TEER) has demonstrated promising outcomes in high-risk patients [8] and likely will continue to be evaluated in other risk groups.

We have previously seen the expansion of transcatheter technologies from prohibitive surgical risk patients to low-risk patients with transcatheter aortic valve replacements [9, 10]. TEER may be following a similar path, having been approved for use in prohibitive-risk patients in 2013 [11] and its utilization in intermediate-risk patients currently being evaluated by the REPAIR MR Study (NCT04198870) [12] comparing the outcomes of TEER against surgical MVP. With the prospect of future clinical trials evaluating the use of TEER in low-risk patients, understanding the contemporary outcomes of MVP, especially in the low-risk group, is paramount to understand if there is a role for transcatheter devices. The aim of this study is to examine the observed versus expected Society of Thoracic Surgeons (STS) morbidity and mortality [13] for low- and non-low-risk patients with primary MR who underwent MVP over the last 15 years. We also identified patients of a very low-risk category that may have the maximal benefit with the highest procedural safety for our analyzed outcomes.

## PATIENTS AND METHODS

### Ethical statement

This study was approved by the Mass General Brigham Institutional Review Board with waived informed consent (Protocol Number 2010P000292, initial approval date 9 February 2010).

### Study population and design: retrospective cohort study

From January 2005 and December 2018, all patients with a diagnosis of primary MR who underwent isolated MVP, or MVP with concomitant tricuspid valve repair or Maze procedure, at a single institution were included in the study. Patients with a diagnosis of rheumatic mitral valve disease, secondary MR, active infective endocarditis, prior cardiac surgery and those undergoing other concomitant cardiac procedures were excluded. Patients were then stratified into 2 risk categories based on age and STS Predicted Risk of Mortality (PROM) scores. For each patient, the STS-PROM was calculated based on the published STS risk model for isolated MVP [13, 14] at the time the patient underwent surgery. Patients with an STS-PROM ≤2% and age ≤75 years were characterized as ‘low-risk’ and patients with an STS-PROM > 2% or age > 75 years were characterized as ‘non-low risk’. The STS-PROM and age cut-offs for the non-low-risk group were congruent with the definition of intermediate-risk patients and inclusion in the REPAIR MR trial [12]. Because the patient population was heavily skewed toward low risk, a subgroup of ‘very low-risk’ patients were defined as having STS-PROM <1% and age ≤75 years. In analyses including the very low-risk group, the definition of low-risk was adjusted to STS-PROM ≥1% but ≤2% and age ≤75 years.

### Primary and secondary outcomes

Primary outcomes included the observed values and observed-to-expected (O:E) ratios for operative mortality and composite morbidity and mortality. Composite morbidity and mortality was defined as the occurrence of any one of the following: operative mortality, stroke, renal failure, prolonged intubation, mediastinitis/deep sternal wound infection or reoperation. Secondary outcomes included stroke, renal failure, prolonged intubation, mediastinitis/deep sternal wound infection, reoperation and length of stay. The definitions for these outcomes were identical to the STS definitions used in their risk prediction models and are found in Supplementary Material, Table S1 [13, 14].

The observed values for these outcomes were obtained on chart review. The expected values for each of the STS outcomes were calculated for each individual patient with their preoperative comorbidity burdens using the STS model that was applicable for the year the patient was operated on. Over the course of the study, STS versions 2.5, 2.6, 2.73, 2.81 and 2.9 were used. O:E ratios were calculated by dividing the incidence of the observed outcome by the mean expected incidence for each outcome.

### Statistical analysis

Continuous variables were tested for distribution and compared using *T*-tests for normally distributed variables and Wilcoxon rank sum or Kruskal–Wallis tests if non-normally distributed. These are presented as mean and standard deviation or median and interquartile range as appropriate. Categorical variables are presented as number and percentage and were compared using Fisher’s exact test. Analyses compared the observed outcomes, expected outcomes, as well as O:E ratios for each of the 7 STS outcomes for the low- and non-low-risk groups. Kaplan–Meier cumulative survival analysis was also performed as a supplemental analysis and is provided within the Supplementary Material, Appendix.

To examine the trends of observed and expected outcomes over time, the observed rate and the mean STS risk score, along with 95% confidence intervals for operative mortality and composite morbidity and mortality, were calculated for each quarter (3 months) during the study period resulting in data points for a total of 56 quarters. The upper and lower confidence limits for expected rates and the mean rate of observed outcomes were plotted, and interpolation lines calculated. This allowed for the comparison of our observed rate to expected rates while presenting any changes over time in patient complexity and expected outcomes. The trend in the mean rate of observed outcomes was then compared to the overall rate for the specific outcome, to evaluate the absolute change in postoperative morbidity and mortality over time.

All analyses were conducted using SPSS version 26.0 (IBM Corporation, Armonk, NY, USA) or R version 3.4.1 (R Foundation, Vienna, Austria). A two-sided *P*-value of ≤0.05 was the criterion for significance for all statistical tests. Data were analyzed from October 2020 to April 2021.

## RESULTS

### Characteristics of patients undergoing MVP

A total of 1207 patients were included in the study. Of those patients, the mean age was 59.9 years, 37.7% were female and 93.5% had moderate or greater MR. The overall STS-PROM was 0.84% and the predicted STS composite morbidity and mortality was 9.43%.

When stratified by surgical risk, 1053 patients were characterized as low risk and 154 patients were characterized as non-low risk. The non-low-risk patients were significantly older (78.5 vs 57.2 years) and more likely to be female (50.0% vs 35.9%) and had a significantly higher mean STS-PROM (2.93% vs 0.54%) (all *P* < 0.001) compared to the low-risk patients. Full patient characteristics and predicted outcomes are presented in Table 1.

**Table 1:** Baseline characteristics of patients undergoing mitral valve repair

	Overall (*N* = 1207)	Low risk (*N* = 1053)	Non-low risk (*N* = 154)	*P*-Value
Age, mean (SD)	59.9 (12.1)	57.2 (10.2)	78.5 (5.7)	**0.001**
Females	455 (37.7%)	378 (35.9%)	77 (50.0%)	**0.001**
Hypertension	540 (44.7%)	428 (40.6%)	112 (72.7%)	**0.001**
Renal failure on dialysis	12 (1.0%)	4 (0.4%)	8 (5.2%)	**0.001**
Congestive heart failure	340 (28.2%)	255 (24.2%)	85 (55.2%)	**0.001**
Arrhythmia	101 (8.4%)	71 (6.7%)	30 (19.5%)	**0.001**
NYHA class III/IV	252 (20.9%)	190 (18.0%)	62 (40.3%)	**0.001**
Prior CABG	6 (0.5%)	2 (0.2%)	4 (2.6%)	**0.003**
Prior valve surgery	0 (0%)	0 (0%)	0 (0%)	–
Mitral stenosis^a^	16 (1.3%)	15 (1.4%)	1 (0.6%)	0.17
Mitral regurgitation^a^				0.13
Moderate	122 (10.1%)	105 (10.0%)	17 (11.0%)	
Severe	1007 (83.4%)	886 (84.1%)	121 (78.6%)	
Tricuspid regurgitation^a^				**0.001**
Moderate	132 (10.9%)	88 (8.4%)	44 (28.6%)	
Severe	20 (1.7%)	7 (0.7%)	13 (8.4%)	
Presenting cardiogenic shock	3 (0.2%)	0 (0%)	3 (1.9%)	**0.002**
Predicted STS risk, mean % risk (SD)				
Mortality	0.84% (1.10%)	0.54% (0.35%)	2.93% (1.82%)	**0.001**
Deep sternal wound infection	0.15% (0.09%)	0.14% (0.08%)	0.22% (0.11%)	**0.001**
Reoperation	5.25% (1.72%)	4.83% (1.17%)	8.19% (2.06%)	**0.001**
Stroke	0.90% (0.55%)	0.75% (0.34%)	1.91% (0.65%)	**0.001**
Prolonged ventilation	4.62% (3.38%)	3.81% (1.60%)	10.30% (6.09%)	**0.001**
Renal failure	1.75% (1.93%)	1.26% (0.80%)	5.14% (3.51%)	**0.001**
Composite mortality and morbidity	9.43% (5.19%)	8.03% (2.78%)	19.24% (7.19%)	**0.001**
Short stay (<6 days)	55.72% (16.17%)	60.0% (11.9%)	26.0% (9.9%)	**0.001**
Long stay (>14 days)	3.50% (3.02%)	2.7% (1.4%)	9.3% (4.6%)	**0.001**

Low risk: patients with a Society of Thoracic Surgeons Predicted Risk of Mortality ≤2% and age ≤75 years; non-low risk: patients with a Society of Thoracic Surgeons Predicted Risk of Mortality >2% and age >75 years. All values are [*N* (%)] unless otherwise specified.

a Degree of valvular disease is based on transthoracic echo values.

CABG: coronary artery bypass graft; NYHA: New York Heart Association; SD: standard deviation; STS: Society of Thoracic Surgeons.

The Bold values represents, if the associated p-value is <0.05 - which was representative of statistical significance in our study.

### Observed intraoperative characteristics and postoperative outcomes

Overall, 94.8% of procedures were elective, 14.7% had a concomitant Maze and 6.5% had concomitant tricuspid valve repair. Overall mortality was low at 0.7% and composite morbidity and mortality was 6.6%. The most common morbidity was prolonged ventilation (3.6%), with stroke being the second most common (1.7%).

Examining by patient risk group, the non-low-risk patients had a significantly higher proportion of urgent and emergent procedures. The low-risk group had significantly lower incidences of mortality (0.2% vs 4.5%), stroke (1.2% vs 4.5%), prolonged ventilation (2.9% vs 8.4%), renal failure (0.6% vs 2.6%) and composite morbidity and mortality (5.1% vs 16.9%; all *P* < 0.05) compared to the non-low-risk group. There was no significant difference in reoperation or 30-day readmission. Full intraoperative characteristics and postoperative outcomes are presented in Supplementary Material, Table S2 and Table 2, respectively.

**Table 2:** Intraoperative characteristics and postoperative outcomes for patients undergoing mitral valve repair

	Overall (*N* = 1207)	Low risk (*N* = 1053)	Non-low risk (*N* = 154)	*P*-Value
Procedure status				**0.001**
Elective	1144 (94.8%)	1006 (95.5%)	138 (89.6%)	
Urgent	60 (5.0%)	47 (4.5%)	13 (8.4%)	
Emergent	3 (0.2%)	0 (0%)	3 (1.9%)	
Postoperative outcomes				
Hours in ICU, median [IQR]	41.5 [24, 54]	35 [23, 51]	50.5 [39, 93]	**0.001**
Length of stay (days), median [IQR]	6 [5, 8]	5 [5, 7]	8 [6, 10]	**0.001**
New-onset atrial fibrillation	242 (20.0%)	194 (18.4%)	48 (31.2%)	**0.001**
Mortality	9 (0.7%)	2 (0.2%)	7 (4.5%)	**0.001**
Deep sternal wound infection	3 (0.2%)	0 (0%)	3 (1.9%)	–
Reoperation	16 (1.3%)	13 (1.2%)	3 (1.9%)	0.26
Overall stroke	20 (1.7%)	13 (1.2%)	7 (4.5%)	**0.02**
Major stroke	12 (1.0%)	7 (0.7%)	5 (3.2%)	**0.01**
Prolonged ventilation	44 (3.6%)	31 (2.9%)	13 (8.4%)	**0.002**
Renal failure	10 (0.8%)	6 (0.6%)	4 (2.6%)	**0.03**
Composite mortality and morbidity	80 (6.6%)	54 (5.1%)	26 (16.9%)	**0.001**
Discharged home	1063 (88.1%)	979 (93.0%)	84 (54.5%)	**0.001**
30-Day readmission	120 (9.9%)	106 (10.1%)	14 (9.1%)	0.83

Low risk: patients with a Society of Thoracic Surgeons Predicted Risk of Mortality ≤2% and age ≤75 years; non-low risk: patients with a Society of Thoracic Surgeons Predicted Risk of Mortality >2% and age >75 years. All values are [*N* (%)] unless otherwise specified.

ICU: intensive care unit; IQR: interquartile range.

The Bold values represents, if the associated p-value is <0.05 - which was representative of statistical significance in our study.

There was no significant difference in cumulative survival between the low- and non-low-risk groups (*P* = 0.34), Kaplan–Meier survival curves are presented in Supplementary Material, Fig. S1.

### O:E ratios of patients undergoing MVP

For the low-risk patients, the observed outcomes were better than expected (O:E ratio <1) for mortality (0.4), deep sternal wound infection (0), reoperation (0.3), prolonged ventilation (0.8), renal failure (0.5) and composite morbidity and mortality (0.6). Observed major stroke (defined as postoperative stroke with residual deficits requiring rehabilitation or resulting in mortality) was lower than expected with O:E ratio 0.9, although overall stroke rate was higher than expected with O:E 1.9. For the non-low-risk patients, observed outcomes were better than expected for reoperation (0.2), prolonged ventilation (0.8), renal failure (0.5) and composite morbidity and mortality (0.9). However, the observed incidence of mortality (O:E ratio 1.5), deep sternal wound infection (8.8), overall stroke (2.4) and major stroke (1.7) were worse than expected. The full observed and expected outcomes, as well as O:E ratios, are presented in Table 3.

**Table 3:** Observed versus expected society of thoracic surgeons outcomes for patients undergoing mitral valve repair

	Observed, *N* (%)			Expected (%)			Observed-to-expected ratio	
	Low risk	Non-low risk	*P*-Value	Low risk	Non-low risk	*P*-Value	Low risk	Non-low risk
Mortality	2 (0.2%)	7 (4.5%)	**0.001**	0.54	2.93	**0.001**	0.4	1.5
Deep sternal wound infection	0 (0%)	3 (1.9%)	–	0.14	0.22	**0.001**	0.0	8.8
Reoperation	13 (1.2%)	3 (1.9%)	0.26	4.83	8.19	**0.001**	0.3	0.2
Stroke	13 (1.2%)	7 (4.5%)	**0.02**	0.75	1.91	**0.001**	1.9	2.4
Prolonged ventilation	31 (2.9%)	13 (8.4%)	**0.002**	3.81	10.3	**0.001**	0.8	0.8
Renal failure	6 (0.6%)	4 (2.6%)	**0.03**	1.26	5.14	**0.001**	0.5	0.5
Composite mortality and morbidity	54 (5.1%)	26 (16.9%)	**0.001**	8.03	19.24	**0.001**	0.6	0.9

Low risk: patients with a Society of Thoracic Surgeons Predicted Risk of Mortality ≤2% and age ≤75 years; non-low risk: patients with a Society of Thoracic Surgeons Predicted Risk of Mortality >2% and age >75 years.

The Bold values represents, if the associated p-value is <0.05 - which was representative of statistical significance in our study.

### Subgroup analyses—very low-risk group

A subgroup analysis was conducted examining a very low-risk subgroup (STS-PROM <1% and age ≤75 years) that consisted of 936 patients compared to low-risk and non-low-risk patients. The very low-risk group had a mortality O:E ratio of 0 compared to the new low-risk group of 1.31. The low O:E values of the low-risk group were mainly driven by the superior outcomes in the very low-risk group—full results of the very low-risk subgroup analysis are presented in Table 4.

**Table 4:** Observed versus expected outcomes following mitral valve repair with very low-risk subgroup

	Observed, *N* (%)			Expected (%)			Observed-to-expected ratio		
	Very low risk (*N* = 936)	Low risk (*N* = 117)	Non-low risk (*N* = 154)	Very low risk (*N* = 936)	Low risk (*N* = 117)	Non-low risk (*N* = 154)	Very low risk (*N* = 936)	Low risk (*N* = 117)	Non-low risk (*N* = 154)
Mortality	0 (0%)	2 (1.7%)	7 (4.6%)	0.44	1.31	2.93	0	1.31	1.55
Deep sternal wound infection	0 (0%)	0 (0%)	3 (2.0%)	0.13	0.22	0.22	0	0	8.80
Reoperation	11 (1.2%)	2 (1.7%)	3 (2.0%)	4.63	6.55	8.19	0.25	0.26	0.24
Stroke	14 (1.5%)	1 (0.9%)	7 (4.6%)	0.69	1.33	1.91	2.18	0.64	2.38
Prolonged ventilation	23 (2.5%)	8 (6.8%)	13 (8.4%)	3.46	6.84	10.30	0.71	1.00	0.82
Renal failure	3 (0.3%)	3 (2.6%)	4 (2.6%)	1.08	2.80	5.14	0.30	0.92	0.51
Composite mortality and morbidity	45 (4.8%)	9 (7.7%)	26 (16.9%)	7.39	13.42	19.24	0.65	0.57	0.88

Very low risk: patients with a Society of Thoracic Surgeons Predicted Risk of Mortality <1% and age ≤75 years; low risk: patients with a Society of Thoracic Surgeons Predicted Risk of Mortality ≥1% but ≤2% and age ≤75 years; non-low risk: patients with a Society of Thoracic Surgeons Predicted Risk of Mortality >2% or age >75 years.

## DISCUSSION

**Figure 1:**
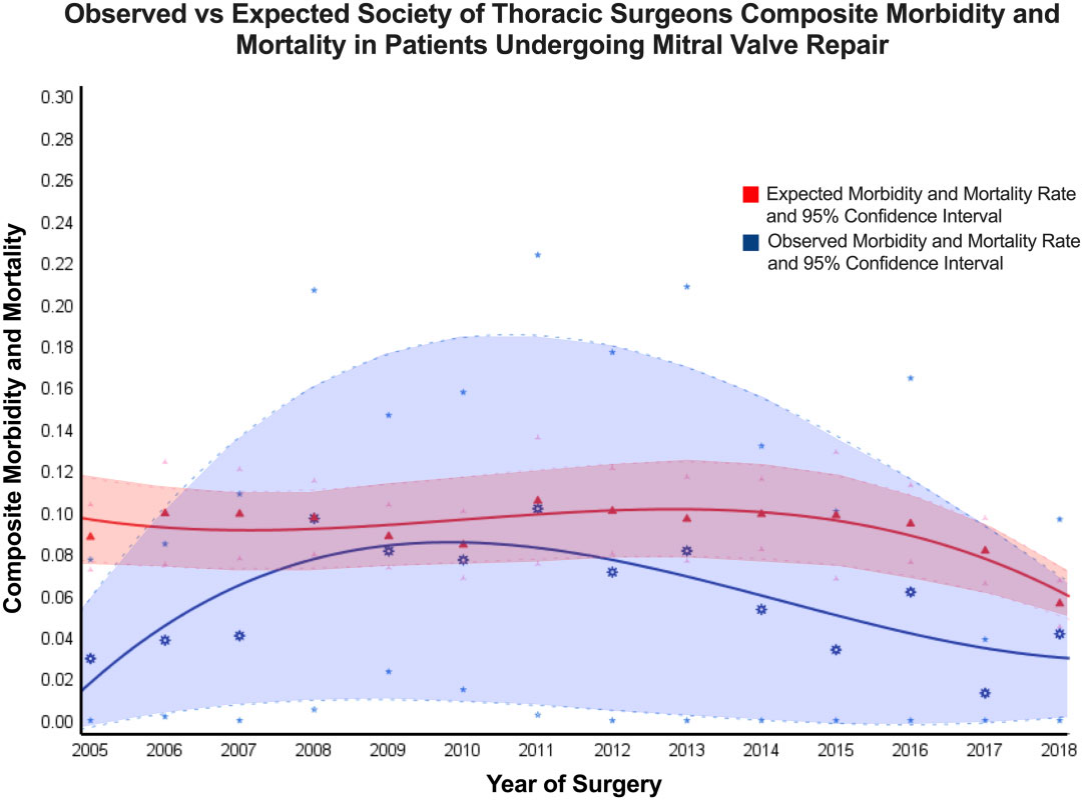
Trends in observed and expected values for composite morbidity and mortality following mitral valve repair. The observed and expected values by quarter for the Society of Thoracic Surgeon’s Composite Morbidity and Mortality outcome are plotted from 2005 to 2018. The red triangles are expected values, red line is best fit of expected values and red shading shows the 95% confidence intervals for expected values. The blue asterisks are the observed values, blue line is best fit of observed values and blue shading shows the 95% confidence intervals for observed values. The observed values are below the lower bound of the 95% confidence interval of the expected values, and trend below the study’s mean observed rate of 6.63% in the latter half of the study period.

In this longitudinal study that examined the observed and expected morbidity and mortality following MVP in low-risk patients, we report several significant findings. First, for low-risk patients, the observed outcomes following MVP are better than expected for mortality and composite morbidity and mortality. Second, the observed rate of composite morbidity and mortality has trended down over time, staying below the 95% confidence interval for expected values in the latter half of the study (Fig. 1). Finally, the excellent outcomes seen in the low-risk group were driven by the very low-risk subgroup where the O:E ratio for mortality was 0. This report emphasizes that low-risk patients have excellent outcomes following MVP, and contemporary outcomes for this group should be analyzed nationally to determine if future transcatheter devices have any role in low- and very low-risk patients.

The low-risk patients in our study had excellent outcomes with a mortality rate of 0.2% and composite morbidity and mortality rate of 5.1%. MVP has had a decreasing mortality rate over the last several decades, with recent studies demonstrating operative mortality rates of 1.1–3.5% [4, 15, 16]. Despite the growth of mitral valve surgery for primary MR [4], there is a paucity of literature looking at the outcomes for low- and very low-risk patients. In our study, the overall mean rate of composite morbidity and mortality was 6.6% and trended down over the course of the study– with observed values largely remaining below expected values (Fig. 1). Accurate benchmarking of contemporary outcomes for MVP by risk group will be vital to safely evaluate the appropriate use of transcatheter technologies that are on the horizon. TEER has demonstrated promising results in high-risk patients with reported in-hospital mortality rates of 2.7% [8]. The REPAIR MR [12] trial is currently evaluating its use in intermediate-risk patients (which corresponds to the same minimum STS-PROM and age cutoffs as our study’s non-low-risk group). The diffusion from high-risk to low-risk patients has been previously seen with transcatheter aortic valve devices and future clinical trials will likely compare the outcomes of low-risk MVP to TEER. Our study provides strong caution, as the excellent outcomes for MVP in the low-risk group will be the gold standard even from the safety perspective, which future studies should examine on a national level. The efficacy of eliminating MR is higher with surgical MVP than TEER from previous reports [8, 17], which gives MVP further advantage in younger patients.

Due to our study population’s bias towards the low-risk group, we identified a very low-risk subgroup (STS-PROM <1% and age ≤75 years) that had 0% mortality and 4.8% composite morbidity and mortality, with O:E ratios 0 and 0.65, respectively. This demonstrates that the excellent outcomes seen in the low-risk group were mainly driven by the very low-risk subgroup. In our study, the original low-risk group had similar O:E patterns as the non-low-risk group (O:E 1.31 and 1.55 for mortality respectively, compared to 0 in very low risk), which questions the use of STS-PROM of 2% as the cutoff for intermediate-risk population. With higher comorbidity burdens, longer lengths of stay and worse than predicted outcomes, we believe that there will be a role for transcatheter interventions within this non-low-risk group. TEER has progressively demonstrated acceptable outcomes for decreasing risk groups—from the EVEREST II trial (minimum STS-PROM = 12%) [11], the COAPT trial (mean STS-PROM = 8.17%) [18] and recent data from the 2013–2015 STS/American College of Cardiology Transcatheter Valve Therapy Registry (mean STS-PROM = 6.1%) [8]. These risk profiles are expected to decrease further, but our study showed that lower-risk groups must have an extremely high standard to apply transcatheter technology, and future trials involving low-risk patients should consider further stratification into very low-risk and low-risk groups.

It is important to discuss stroke, which was the only postoperative morbidity that demonstrated worse than expected outcomes for all risk groups. The overall observed stroke rate of 1.7% in our study is similar to what has been previously reported [16, 19]. Our series, when stratified into major stroke (mortality or persistent symptoms requiring rehabilitation) versus minor stroke (symptoms resolved or persistent symptoms, but patient was discharged home with no services), major stroke was only worse than expected for the non-low-risk group (non-low-risk O:E = 1.7, low-risk O:E = 0.9). Our intensive care unit is a closed unit, and we have a lower threshold for neurology assessment as a multidisciplinary team assessment for any neurological changes, which may explain the higher incidence of minor strokes. In addition, because the observed rate of stroke is similar to what has been reported in other studies, the elevated O:E ratio may be due to the expected values being low for these patients. As this study is based out of single institution, it is also possible that the low frequency of the outcomes combined with the relatively small sample size may lead to the O:E ratios not being statistically different from national benchmarks; however, with the current methodology for how the expected outcomes were calculated, we cannot statistically compare our stroke O:E to a larger cohort and requires further study.

The landscape of cardiac surgery has shifted in the last several decades with the advent of transcatheter technologies for coronary revascularization, aortic replacements and valve replacements/repairs. The careful evaluation of the risks and benefits of these new technologies compared to their surgical gold standards is of the utmost importance. As TEER continues to explore studies looking at lower-risk patients, the observed outcomes following MVP for each risk group should be the benchmark for acceptable postoperative outcomes. Our study’s excellent outcomes of MVP in low-risk, especially the very low-risk patients, indicate that future transcatheter technology must be trialed cautiously and benchmarked against contemporary national outcomes for these groups before further dissemination.

### Limitations

There are several limitations in this study that need to be addressed. This study is a single institution, retrospective, observational study and has the associated limitations of such that include but are not limited to small sample size, selection bias, limited generalizability and unmeasured confounding. In addition, this study is based out of a quaternary, academic care centre that is a comprehensive valve centre, which limits the generalizability of these results to experienced institutions and may not reflect national outcomes. Second, although STS publishes modifiers for PROM adjustment, the scores used in this analysis did not use these modifiers. However, 5 different versions of STS (2.5, 2.6, 2.73, 2.81 and 2.9) were used for expected risk calculation to account for the improvement in MVP outcomes over time. Finally, our study population was limited to patients undergoing MVP and not limited to patients who were intended for MVP and ultimately underwent replacement, which could introduce a selection bias. Nonetheless, this is one of the first studies to examine the observed vs expected morbidity and mortality following MVP and provides valuable contemporary outcomes for future transcatheter devices.

## CONCLUSION

The observed mortality and composite morbidity and mortality following MVP were better than expected for low-risk patients, particularly for the very low-risk groups that demonstrated excellent outcomes. With the future expansion of transcatheter technologies into the intermediate- and potentially low-risk groups, future studies on the national, contemporary outcomes of MVP should be the benchmark for ongoing and upcoming transcatheter device trials. While there appears to be a role for transcatheter devices in improving postoperative outcomes for non-low-risk patients, careful evaluation and the cautious study of this technology in lower-risk groups will be exceedingly critical for patient care.

## SUPPLEMENTARY MATERIAL

Supplementary material is available at *ICVTS* online.

## Funding

This research did not receive any additional funding.

**Conflict of interest:** Tsuyoshi Kaneko is a consultant for Edwards Life Sciences, Medtronic, Cook Medical, 4C Medical and CardioMech and a speaker for Abbott and Baylis. Sameer Hirji is a consultant for Cardiac ERAS Interactive Audit System (EIAS) for ENCARE. The other authors report no conflicts of interest.

## Supplementary Material

ivac241_Supplementary_Data

## Data Availability

The aggregate data underlying this article are available in the article and in its online supplementary material. The complete, individual data points underlying this article will be shared on reasonable request to the corresponding author after de-identification and approval from the Mass General Brigham Institutional Review Board.

## Notes

Presented at the American Association of Thoracic Surgery Annual Meeting, Virtual, 30 April 2021.

## ABBREVIATIONS

MR: Mitral regurgitation
MVP: Mitral valve repair
O:E: Observed-to-expected
PROM: Predicted Risk of Mortality
STS: Society of Thoracic Surgeons
TEER: Transcatheter edge-to-edge repair
