# Supplementary material for: Observed versus expected morbidity and mortality in patients undergoing mitral valve repair
Source: Interact Cardiovasc Thorac Surg. 2022 Oct 7;35(5):ivac241. doi: 10.1093/icvts/ivac241 (PMC9553224; doi:10.1093/icvts/ivac241)
Supplement: ivac241_Supplementary_Data [file ivac241_supplementary_data.docx]

**Supplemental Appendix**

***Supplemental Table 1 – Definition of Primary Outcomes***

| **STS Outcome Endpoint** | **Definition** |
| --- | --- |
| **Operative Mortality** | All cause in-hospital mortality even after 30 postoperative days, or all cause mortality within 30 postoperative days if discharged |
| **Stroke** | Acute episode of focal or global neurologic dysfunction caused by brain, spinal cord, or retinal vascular injury as result of hemorrhage or infarction in which neurologic dysfunction lasts longer than 24 hours |
| **Renal Failure** | New requirement for dialysis or meeting RIFLE criteria based on increasing creatinine levels or decreasing GFR |
| **Prolonged Intubation** | Prolonged intubation or reintubation lasting more than 24 hours |
| **Mediastinitis/Deep Sternal Wound Infection** | Mediastinitis or a deep sternal wound infection occurring during index hospitalization or within 30 postoperative days |
| **Reoperation** | Reoperation for bleeding, tamponade, or any cardiac reason |
| **Composite Morbidity and Mortality** | A composite score defined as the occurrence of any one or more of the defined endpoints above |

**Supplemental Table 2 - *Intraoperative Characteristics for Patients Undergoing Mitral Valve Repair***

|  | Overall  [N=1207] | Low-Risk  [N=1053] | Non-Low Risk  [N=154] | P-Value |
| --- | --- | --- | --- | --- |
| Operative Approach |  |  |  |  |
| *^a^Full Sternotomy* | 733 (60.7%) | 608 (57.7%) | 125 (81.2%) | **<0.001** |
| *Partial Sternotomy* | 359 (29.8%) | 338 (32.1%) | 21 (13.6%) | **<0.001** |
| *Mini Right Anterior Thoracotomy* | 80 (6.6%) | 74 (7.0%) | 6 (3.9%) | **0.03** |
| *Right Anterior Thoracotomy* | 19 (1.6%) | 19 (1.8%) | 0 (0%) | - |
| *Other* | 4 (0.3%) | 4 (0.4%) | 0 (0%) | - |
| *Missing/Not Documented* | 12 (1.0%) | 10 (0.9%) | 2 (1.3%) | - |
| Intraoperative Details | | | | |
| *Concomitant Maze* | 178 (14.7%) | 147 (14.0%) | 31 (20.1%) | 0.07 |
| *Concomitant Tricuspid Valve Repair* | 79 (6.5%) | 48 (4.6%) | 31 (20.1%) | **0.001** |
| *Perfusion Time, min [Median, IQR]* | 117 [96,145] | 117 [97,145] | 114 [94,140] | 0.27 |
| *Cross Clamp Time, min [Median, IQR]* | 83 [68, 104] | 83 [68, 105] | 80.5 [64, 101] | 0.18 |
| Mitral Valve Pathology |  |  |  | 0.72 |
| *Myxomatous/Prolapse* | 1,006 (83.3%) | 874 (83.0%) | 132 (85.7%) |  |
| *Barlow* | 107 (8.9%) | 97 (9.2%) | 10 (6.5%) |  |
| *Cleft* | 3 (0.2%) | 3 (0.3%) | 0 (0%) |  |
| *Congenital* | 3 (0.3%) | 3 (0.3%) | 0 (0%) |  |
| *Healed Endocarditis* | 4 (0.3%) | 3 (0.3%) | 1 (0.6%) |  |
| *Ruptured Chord* | 9 (0.7%) | 7 (0.7%) | 2 (1.3%) |  |
| *Not Documented/Missing* | 75 (6.2%) | 66 (6.2%) | 9 (5.8%) |  |
| Mitral Valve Procedure Characteristics (N=1,134) |  |  |  |  |
| *Commisuroplasty* | 113 (10.0%) | 88 (8.9%) | 25 (17.5%) | **0.003** |
| *Ring Annuloplasty* | 1,131 (99.7%) | 990 (99.9%) | 141 (98.6%) | **0.04** |
| *Leaflet Resection* | 626 (55.2%) | 567 (57.2%) | 59 (41.3%) | **0.001** |
| *Posterior* | 457 (40.3%) | 416 (42.0%) | 41 (28.7%) | - |
| *Anterior* | 4 (0.4%) | 3 (0.3%) | 1 (0.7%) | - |
| *Both* | 6 (0.5%) | 6 (0.6%) | 0 (0%) | - |
| *Edge-to-Edge* | 104 (9.2%) | 90 (9.1%) | 14 (9.9%) | 0.76 |
| *Sliding Valvuloplasty* | 229 (20.2%) | 221 (21.3%) | 18 (12.6%) | **0.05** |
| *Chordoplasty* | 83 (7.3%) | 71 (7.2%) | 12 (8.4%) | 0.61 |
| *Cleft Repair* | 133 (11.7%) | 106 (10.7%) | 27 (18.9%) | **0.008** |

*IQR=Interquartile Range; Min=Minutes. Low-Risk = Patients with a Society of Thoracic Surgeons Predicted Risk Of Mortality* ≤*2% and age* ≤*75 years; Non-Low Risk = Patients with a Society of Thoracic Surgeons Predicted Risk Of Mortality >2% and age >75 years. ^a^Full sternotomy is reference group for operative approach comparisons. All values are [N(%)] unless otherwise specified.*

**Supplemental Figure 1 – *Cumulative Survival for Low and Non-Low Risk Groups Following Mitral Valve Repair***


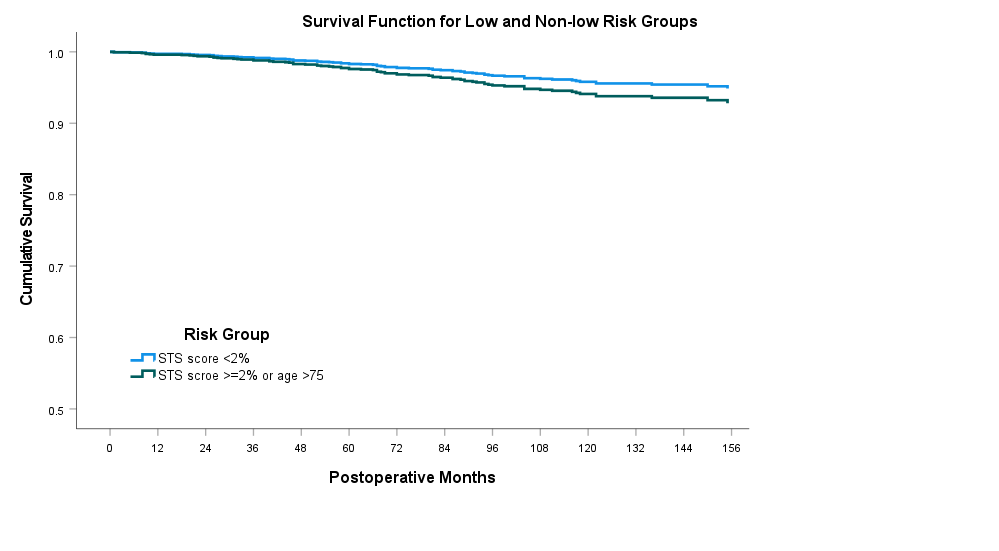


*STS=Society of Thoracic Surgeons.*
